# Selections

**Published:** 1817-09

**Authors:** 


### PART III.

### SELECTIONS.

—— Factorum est copia nobis.

### FOREIGN WORKS.

#### I. ANATOMY.—*Case of Extroversion of the Bladder.**

The subject of this observation is a male child, born September 17th, 1815. The abdominal tumour, of a bright red colour, and

* *Cas d'Extroversion de la Vessie,* par M. le Docteur Voisin. *Bulletin de la Faculté de Médecine de Paris.* No. II.

covered with mucus, presented the volume and nearly the figure of a large almond. It extended from the navel, of which it made a part, and from which the funis had already separated, to the superior border of the pubis, and was longest in this direction. Its surface was villous, and highly sensible. The urine, at first, seemed to ooze from numerous pores; but, two days afterwards, it evidently escaped from two orifices placed parallel to the pubic border of the tumour. The scrotum, with its raphe, was strongly marked; the testicles had descended, but the penis presented an example of *epispadias*, for its urethral surface was uppermost; and, instead of the urethra, was seen a groove commencing at the extremity of the glans, which was cleft by it, and terminating beneath the pubic border of the tumour. The corpora cavernosa existed, but it was impossible to determine whether the penis, in this state, preserved its erectility. To obviate the irritation caused by the acrimony of the urine and sensibility of the tumour, cleanliness, mucilaginous, and saturnine applications with opium, were prescribed.

Four months after birth, the child continued well. The tumour was then constantly very sensible; and tenesmus and an increased flow of urine from the ureteric orifices, were induced by touching it. It presented a quadrangular instead of an oblong form, and an augmentation of volume. The umbilicus could no longer be distinguished. The structure of the mucous membrane was more lax, and copiously smeared with mucus. The umbilical border of the tumour was sixteen lines in extent; and its angles, slightly rounded, were separated by a deep fissure. The pubic border, about an inch in length, was papillated *(mameloné)*: its angles, formed by an elongation of the ureteric orifices, which gave them the size and figure of the teat of a small bitch. The fissure separating them was deep. The borders, corresponding to the iliac regions, were about eight lines in extent.—Dr. Voisin proposes to obviate the inconvenience resulting from this malformation, by the constant use of a small receptacle, formed of elastic gum.

---

### II. PATHOLOGY and PRACTICE. *Case of Ascites.**

THE following history, which will be found to possess great singularity and interest, is detailed by Dr. Bezard. A widow, residing in Paris, had enjoyed good health during her infancy. Menstruation commenced at the age of 15, and continued regular till the period of marriage, which took place about her 25th year. The union was unfortunate. During the three first years, in consequence of blows inflicted on the abdomen, she was thrice prematurely delivered about the seventh month of pregnancy. The effect of these

---

* *Observation sur une hydropisie ascite. Par M. Bezard, D. M. Bulletin de la Société Medicale d'Emulation.* No. xii. Decembre, 1815.

contusions was not confined to the exterior. The woman was soon attacked with deep-seated and obtuse pains about the abdomen succeeded by languor. The menses were suppressed; the abdomen gradually enlarged; and, in the year 1797, dropsy was apparent. All the usual remedies were tried without success, for some months, and, at length, twenty *pintes** of limpid fluid were evacuated by paracentesis. Medicine still proving useless, the operation was repeated at the end of a month. The abdomen afterwards filled so rapidly, that it became necessary to operate more frequently; and from fifteen to twenty *pintes* of water were drawn off every eight days. Towards the close of 1801, the patient came under the care of Dr. Bezard; who suspected some exaggeration until assured by her surgeon, M. Chateauneuf, that the operation of paracentesis had already been performed upon her, one hundred and eighty times.

Every six, seven, or eight days after this, Dr. Bezard continued to operate, and drew away each time, from fifteen to twenty *pintes* of water. On examination after the first puncture, he found a considerable tumour in the umbilical region. To temporize with respect to the operation, as is practicable in many cases of ascites, would not do here. No sooner were the abdominal integuments distended to a certain point, than lancinating and intolerable pains came on: and in the event of Dr. Bezard being from home, the woman, forgetting the natural timidity of her sex, would call for the trocar, and introduce it with her own hand into the abdominal cavity. She uttered dreadful screams during this operation, which commonly required a quarter of an hour to perform. The volume of the abdomen not allowing her to distinguish the point where the instrument ought to be introduced, a neighbour assisted to direct it at hazard among the cicatrices of former operations: consequently considerable hæmorrhage sometimes took place from wounded blood-vessels. Nevertheless, the woman thus operated upon herself more than sixty times.

Still her husband continued his horrible brutality. One day, when Doctor Bezard called to operate, he found the poor woman black from head to foot with bruises. The wretch, however, was at length driven from home by general execration; and fell, in a manner far more honourable than he deserved, on the field of Austerlitz. The woman, rendered tranquil by this event, gave herself

* The French *pinte* is used in the dictionaries as synonymous with the English *pint;* but the *pinte* of old French measure is somewhat more than equivalent to the modern *litre* (about two English wine pints) and, in this sense, it is obviously from the computation at the close of his paper, that the term *pinte*, is employed by *Bezard.* In our recent review of "*The Continental Medical Repertory*", this uncommon history is slightly glanced at; but, on referring to the original in *Leroux's Journal,* we found the particulars of it very inaccurately stated by the Hamburgh Editor; and considered it worthy of a more correct and extended introduction to our readers. EDITORS.

up to the consolations of religion. In 1802, she was urged by her priest to witness one of the grand ceremonies of the Romish church; and, although it was the day on which the operation ought again to have been performed, and her bulk was at that time enormous, she complied. On attempting to pass under the canopy a second time, she fainted. The women who ran to her assistance, found her clothes wet. She was carried home; and, on recovering, saw that the abdomen was sunk, and that the water came away by the urinary passages. In fact, by noon the swelling was gone; and the collection of water had been voided by the urethra. For three months after, the operation of paracentesis was not repeated. The abdomen continued to swell during five or six days; yet, ere it reached that state of tension which had always before rendered puncture necessary, the fluid was evacuated by urine. But nature ceased to favour this salutary process; and the belly soon acquired a bulk, attended with such severe pains as again to require paracentesis. Yet the patient indulged the hope of relief by the urinary passages; and hence used every effort to defer the moment of operation. But this delay served only to alter the direction which nature had at first chosen; and the water, exuding through the digestive organs,* was rejected by vomiting. This extraordinary evacuation was several times repeated; and the fluid, which had always been limpid, was then bloody. Dr. Bezard has seen the woman vomit sixteen *pintes* of it mixed with fæcal matter. She however, soon preferred the more simple process of puncture to this disgusting mode of evacuation; and the operation was regularly performed once a week.

In the year 1808, the *digitalis purpurea* was tried; but, even in doses of half a grain, its effects upon the head were such that it could not be continued. Paracentesis was now the only resource; and nothing farther was attempted. Meanwhile, the weakness was increased. Solid aliment could no longer be swallowed; and sugared water constituted the woman's sole nutriment. Towards the close of life, she took but a few spoonfuls of coffee, so that an ordinary cup of it was sufficient for two days. The vital principle gradually sank away, but the intellectual faculties were unimpaired. At length, the arterial pulsations were nothing more than a slight fluttering beneath the finger; all motion, even of the lips, was impracticable; and respiration was nearly imperceptible. Yet, contrary to the expectation of Dr. Bezard, the woman lingered till August 26th, 1811.

It appeared, upon inquiry, that in the intervals from one operation to another, the woman had never taken a quantity of food, in solids and liquids together, equal to that of the fluid which was drawn from the abdomen. During the progress of the disease,

---

* We profess ourselves unable to comprehend precisely the meaning of this expression. Editors.

which occupied a term of thirteen years, she had suffered once from typhoid fever and dysentery, and twice from pneumonia: yet these affections were successfully treated, without at all interrupting the regular performance of the operation for emptying the abdomen, and the patient continued her ordinary domestic occupations till the close of 1802. On connecting the circumstances of the case throughout, it was found that the woman had six hundred and sixty five times undergone the operation of paracentesis; that the fluid had been twenty times evacuated by the route of either the urinary or digestive organs; and that, from all the operations, taking fifteen *pintes* as the average product of each, there had been obtained a total of ten thousand two hundred and seventy-five *pintes* of fluid, or about thirty-five Burgundy hogsheads (*muids de Bourgogne*).

*Dissection.* Nearly a month had elapsed since the last operation was performed, yet the abdomen was supple, and yielded only a slight fluctuation. About four *pintes* of purulent fluid were drawn off by a trocar: it was of a greyish white colour, had the consistence of milk, and the smell commonly perceptible on the opening of a body. The surface of the abdomen was ash coloured, and the skin was wrinkled in every direction, as happens in all cases where the integuments have suffered great distension: it moreover offered considerable resistance to the scalpel, and adhered so intimately to the abdominal muscles, that its separation was impracticable. The cellular structure consisted of close meshes, which united the muscles and skin so as to form one flattened substance two lines in thickness. The muscular fibres were dried, and strongly adherent to each other. The utmost force was required in dividing with the scalpel the linea alba and peritonæum. The latter membrane was three lines thick and completely cartilaginous. It exhibited, at first sight, the appearance of rind of bacon. On turning aside the abdominal parietes, not the slightest trace of omentum could be discovered. The intestines were seen bare, inflamed, mutually adhering, contracted in diameter, and closely connected with the vertebral column, where they occupied a confined space. The large intestines shared the contraction, and the whole canal was generally so straight as not to admit the extremity of the little finger. There was no mesentery. The stomach also was inflamed, diminished in volume, and contained a brownish mucous fluid. The kidnies, liver, gall-bladder, pancreas, spleen, and urinary bladder no longer existed. The situations in the abdomen, commonly possessed by these viscera, were unoccupied. A thick smooth membrane only was observed, which going off from (the site of) each of the destroyed organs, was prolonged into the right hypochondrium; where, by the union of all, a scirrhous tumour, possessing the figure of the liver, was formed. It was flattened, six inches in width, and weighed about one pound. On opening this tumour, a greyish pus, of nauseous smell, escaped. Amid the destruction or injury of all the viscera, the uterus alone retained its natural form and colour. The thoracic organs displayed nothing peculiar, except diminution of volume. The intellectual faculties having remained unimpaired till the moment of dissolution, the encephalon was not examined.

We find nothing very original or interesting in Dr. Bezard's "*Reflections*" on this most extraordinary case. The "scirrhous tumour" must have diminished considerably before death; as, for several years previously, it appeared to possess the volume of the (adult) head.

---

### *Observations on the Characters and Treatment of Anæmia.**

This term has been employed by many authors. *Lieutaud*, in his practical medicine, gives it to a disease characterized by all the symptoms of general debility; and in which the mass of blood is so much diminished that the vessels are nearly empty. *M. Hallé* has described, under the same name, an affection which attacked epidemically, in 1803, the workmen in the gallery of a coal-mine at Auzain, near Valenciennes. The gallery in question was the only one of all there worked, which was infected; and all the men who laboured in it, were without exception successively attacked. The precaution, subsequently taken to close it, did not preserve any of those who had been there. Many fell ill three or four months after having been subjected to the deleterious influence from which the disease derived its origin. This gallery was, nevertheless, situated, like the others, at one hundred and twenty toises below the surface, and similarly ventilated. Yet it was longer, and, consequently, the renewal of air less easy; nor did the openings, made to favour this process, obviate the pernicious consequences. The temperature there varied from 17° to 22° of Reaumur's thermometer, (70° — 81° Fahr.), and respiration was disordered by it. The air of this gallery was analysed by *M. Liegeard*, Professor of Chemistry at Douai, and found to contain sulphuretted hydrogen gas, with a considerable proportion of carbonic acid. The former, deleterious in its influence on the animal œconomy, appears to have been the most probable cause of the epidemic. Some of the sick workmen had also drank the water which oozes through the mine; and in this, upon chemical examination, the presence of sulphuretted hydrogen gas was detected. This fluid was said to raise boils or blisters on the part with which it came in contact: yet, anteriorly to 1802, no similar disease had ever been observed.

The following were the characters which this affection presented: First attack, violent colics; pains in the bowels and stomach; tight respiration; palpitations, weakness, tumidity of the abdomen; black and green stools. After this state has continued ten days or more, cessation of the abdominal pains; feeble, concentrated, quickened

---

* *Dictionnaire des sciences medicales. Article, Anémie ou Anemuse, Anæmia, deficiency of blood, from α priv. and αιμα, blood.*

pulse; skin discoloured and of a yellow tinge; progression difficult and fatiguing; frequent palpitations; bloated countenance; habitual sweats. This second stage is protracted for several months, or even a year, with emaciation and decay. Lastly, renewal of the early symptoms; dreadful head-ache; frequent faintings; intolerance of light and sound; tension and pains of the abdomen; and purulent stools, followed by speedy death.

A very important distinction may be established between the patients, according to the period of the epidemic at which they were attacked. Those first seized, presented, in general, the symptoms which have just been described; but, afterwards, the sudden invasions by violent colics were no longer remarked; and the disease appeared to assume a more chronic form. It came on by a weakness, which did not, at first, incapacitate the patient for work; and gradually augmented. To this was added head-ache, ringing in the ears, anxiety at the præcordia, palpitations; sometimes costiveness, sometimes diarrhœa. The skin became discoloured, and put on the yellowish tinge peculiar to this singular malady; whence it has got the name of the *yellow disease.* The colour, however, is not that of jaundice, but resembles bees-wax grown yellow by age. Several patients, in addition to this invariable symptom, complained only of constant palpitations of the heart, perceptible through their clothes, even in a state of rest, and felt, as it were, by an echo beneath the summit of the head. Cinchona, camphor, opium, wine, generous diet, and many other remedies had been tried without success.

Four of these workmen were conducted by a physician to Paris, received at the *Hospice de la Faculté,* and placed under the especial direction of M. *Hallé.* On arrival, they had all œdema of the face and superior and inferior extremities; but this was merely the effect of the fatigue of the journey, and dissipated by rest. A wan and yellowish tinge was generally diffused, not only over the skin, but over the interior of the eye-lids, lips, and mouth, and even the tongue. No ramification of the capillary vessels was visible on the conjunctiva or gums; and the superficial veins of the hand and arm had disappeared. There was no sensible obstruction in the abdomen, except that the mesentery felt large though without induration. The pulse was constantly from 90 to 100 in a minute, without sensible heat of the skin. But, at certain times, fever came on, with accelerated pulse, and high cutaneous temperature. There were, moreover, frequent palpitationss, and, even in the absence of these, decided pulsations of the heart against the parietes of the chest; inability to walk, especially up an eminence, without sense of suffocation, yet the thorax was sonorous throughout; frequent moisture of the palms of the hands, and habitual night sweats. Three of these men, notwithstanding, ate with avidity such food as they could relish, and digested it well. But the liquid consistence of the stools, and their unhealthy colour, announced some fault in the digestive process. The urine was natural. Some days after their arrival, a plan of diet was laid down.

It consisted of substantial aliment; meat, commonly roasted, well-baked bread, excellent beer, and good wine. Care was taken, at the same time, to guard them from the influence of depressing moral affections. With these means was combined the use of antiscorbutics, bitters, and cinchona. Mercurial frictions, suggested by particular analogies, were soon abandoned.

Under this treatment, one of the patients, habitually more languid, and having less appetite than the rest, died. Fever, which at first came on every other day, in the course of ten days assumed a continued and severe type. Pains in all the limbs, acute head-ache, hard pulse, heat and dryness of the skin without increase of colour, clean tongue, abdomen swollen and painful, and evident resistance in the region of the liver, were the chief symptoms. After forty-eight hours, the fever subsided; pulse feeble; useless efforts to vomit even with the aid of an emetic draught. Violent oppression, intermission of the pulse, and coldness of the extremities, preceded death. On *Dissection*, at the *Hospice de l'Ecole de Médecine*, before Prof. *Hallé*, the abdomen was found slightly tumefied; the colour of the skin the same as before death; the intestines, particularly the colon, distended; the fat of the omentum and mesentery very yellow; the liver small, supple throughout, its substance soft and unctuous to the touch, and of a light yellow colour, extending to the exterior; the gall-bladder half full of bile, resembling in colour the yolk of egg, and found to contain, on analysis, much coagulable albumen; the spleen small, and unnaturally soft; the stomach half filled with a fluid resembling wine-lees, with which the duodenum and jejunum were also coloured; the other abdominal viscera sound. The right lung adhered almost universally to the costal pleura. Both of them yielded, on incision, a yellowish frothy serum, which oozed from all points of their substance, and not from any particular collection. The substance of the heart was as pale as though it had been washed and macerated; its parietes flabby, and columnæ carneæ wasted. Not one drop of red blood flowed from its cavities. A coagulum, as pale as the flesh of the heart, was found in the left ventricle. The brain was white, and nearly of the same shade. More than a drachm of serum was contained in the posterior part of the left lateral ventricle. The plexus choroides was of a pale red colour. In all three cavities, the arterial and venous trunks were generally destitute of coloured blood, and contained only a little serous fluid. No blood was found in the aorta to its crural bifurcation; in the axillary arteries to their brachial subdivisions; in the accompanying veins; in the system of hepatic vessels; nor in any sinus of the brain. On making deep incisions into the muscular substance of the thigh, a small quantity of black and fluid blood escaped; but none from any other part. The thoracic muscles were of a somewhat red colour; those of the extremities less so. This deficiency of blood was constantly found in the dissections performed on the spot, where the disease had broken out.

This phænomenon has been regarded by Hallé as a state essentially dependent on the disease, following all the periods of it, and attaining its acme when the latter is near a close. He also discovered that mercurials ought not to be employed in its treatment; and immediately substituted for them iron-filings, in the dose of one drachm a day, combined with an equal quantity of cinchona and muriate of ammonia. The latter ingredient, as inducing occasional pains in the bowels, was afterwards suppressed.

In about a week the beneficial effects of the new treatment were perceptible. The veins of the arm and palmar surface of the wrist again became prominent. The power of ascending the hospital-stairs, without sense of suffocation, was restored. Soon afterwards the increase of colour was more general; the appetite and digestion improved. Suspension however of the treatment was followed by relapse. But the men, after three months' continuance of it, having regained their health, though not their natural degree of colour, returned home. One of them perished from an accident on the road. The other two were perfectly restored.

While *Hallé* obtained at Paris these fortunate results from the use of chalybeate preparations, Dr. *Lebleu* had derived equal advantages from them in the treatment of four other patients. At first, he employed the red oxyde of iron; but, perhaps fortunately, was afterwards obliged to have recourse to the black oxide and filings.

These experiments have since been turned to good account. The directors of the mines of Auzain cause the workmen, on the first signs of weakness, to discontinue their labours, and give them iron filings mixed in chocolate. Thus the workman is soon enabled to resume his occupation. Yet the restoration is rarely perfect when the affection has been severe; and labour speedily renews the half extinct symptoms of debility. Iron may therefore, be regarded as the antidote of this disease, more exclusively than of chlorosis, which is also a species of anæmia.

The combination of tonics, bitters, and antiscorbutics is certainly useful; although alone, they are clearly inefficient. Generous diet is not less necessary. *Lieutaud*, it may be observed, mentions steel, among the remedies for anæmia; but its efficacy is more fully established by the preceding observations.

---

### *Sanguineous Phthisis cured by Charcoal Powder.**

C. P. aged seventeen years, was admitted into the clinical ward on the 9th of June. She is of a delicate constitution, but had

---

* *Floride Lungensucht durch Kohlenpulver geheilt, beobachtet u. beschrieben;* von Hrn D. Woyde. *Journal der Practischen Heilkunde,* von Hufeland, November 1815.—Our readers have, probably, heard something of the German treatment of phthisis by charcoal powder. What we have seen of its effects is little in favour of the practice. From the sample been formerly free from any serious complaint. She menstruated regularly without difficulty. Some months before, she had suffered from pneumonia, consequent on a severe cold; for which, however, nothing was done. The affection declined, but from this time she had pains in the breast, particularly in the left side; cough, with mucous expectoration, most urgent in a morning; frequent alternations of shivering and heat; and flushings in the hollow of the hands and feet after her meals. The countenance was very red. There were also a sense of increased heat towards evening, night sweats, lassitude, and head-ach. The appetite was irregular; the pulse frequent and somewhat weak.

Six ounces of blood were drawn from the left arm; a blister laid on the painful part of the breast; a powder of calomel, digitalis, and liquorice-root, administered morning and evening; and a solution of muriate of ammonia, with extract of liquorice, every two hours. By these means, the cough and pain in the breast were considerably diminished, and the respiration relieved. There was no change in the pulse and expectoration. As soon as the first appearances of salivary affection were observed, the calomel was discontinued; and, in addition to the ammoniacal solution, were prescribed the hordeum præpar. and digitalis, three times a day. The appetite, which had been nearly lost, now returned; the head-ach had disappeared, but the pains in the breast and the pulse continued the same. On the 23d of June, the patient got, in addition to the digitalis powders, one scruple of recently prepared charcoal powder three times a day. The immediate consequence of this was, that the pain of the breast was very considerably aggravated; the cough stronger, but the expectoration, on the contrary, was lessened; and the pulse continued invariably frequent as before. From June 30th to July 9th, the patient exhibited febrile excitement, accompanied with loss of appetite and constipation of the bowels. A mixture of sal. mirabile (sulphate of soda) with tamarind pulp and antimonial wine, removed this latter symptom, restored the appetite, and somewhat eased the breast. On the 10th of July, the pains in the breast returned; the cough and expectoration were increased; the pulse very frequent. A blister was now laid upon the breast, and kept permanently discharging. Meanwhile, a powder of charcoal, digitalis, and liquorice, was given three times a day, and the hordeum præpar. continued. The patient persevered, without intermission, in these powders; and the dose of charcoal was subsequently raised to four scruples, and that of the digitalis to four grains in a day.

---

now presented to him, the English practitioner will be enabled to draw his own conclusions. We, however, have not only our doubts whether the case here described were really one of phthisis; but whether the cure, whatever the disease were, be not more fairly attributable to the operation of the bleeding, calomel, and digitalis than to that of Dr. Woyde's "*carbo tiliæ recens paratus.*" Edit.

The condition of the patient was now sensibly ameliorated. In July, the pains of the breast gradually subsided. The cough was relieved, but the expectoration yet considerable: the exhausting night sweats had abated; the pulse was lower; yet, towards evening, there was a sense of increased heat. In August, the evening fever and night sweats completely disappeared. Expectoration took place only in a morning, with slight cough. The respiration became nearly free, and the pulse natural. Some febrile symptoms, probably the consequence of cold, shewed themselves in the beginning of September; but an evacuant diaphoretic treatment speedily restored the patient, and she resumed on the 9th the charcoal with digitalis. Henceforth, all the morbid symptoms disappeared, with the exception of a weight in the breast. The pulse was tranquil; the cough and pain completely removed.

